# Leisure time physical activity and subsequent physical and mental health functioning among midlife Finnish, British and Japanese employees: a follow-up study in three occupational cohorts

**DOI:** 10.1136/bmjopen-2015-009788

**Published:** 2016-01-06

**Authors:** Jouni Lahti, Séverine Sabia, Archana Singh-Manoux, Mika Kivimäki, Takashi Tatsuse, Masaaki Yamada, Michikazu Sekine, Tea Lallukka

**Affiliations:** 1 Department of Public Health, University of Helsinki, Helsinki, Finland; 2 Department of Epidemiology and Public Health, University College London, London, UK; 3 Finnish Institute of Occupational Health, Helsinki, Finland; 4 Department of Epidemiology and Health Policy, University of Toyama, Toyama, Japan

**Keywords:** PREVENTIVE MEDICINE, PUBLIC HEALTH, SPORTS MEDICINE

## Abstract

**Objectives:**

The aim of this study was to examine whether leisure time physical activity contributes to subsequent physical and mental health functioning among midlife employees. The associations were tested in three occupational cohorts from Finland, Britain and Japan.

**Design:**

Cohort study.

**Setting:**

Finland, Britain and Japan.

**Participants:**

Prospective employee cohorts from the Finnish Helsinki Health Study (2000–2002 and 2007, n=5958), British Whitehall II study (1997–1999 and 2003–2004, n=4142) and Japanese Civil Servants Study (1998–1999 and 2003, n=1768) were used. Leisure time physical activity was classified into three groups: inactive, moderately active and vigorously active.

**Primary outcome measure:**

Mean scores of physical and mental health functioning (SF-36) at follow-up were examined.

**Results:**

Physical activity was associated with better subsequent physical health functioning in all three cohorts, however, with varying magnitude and some gender differences. Differences were the clearest among Finnish women (inactive: 46.0, vigorously active: 49.5) and men (inactive: 47.8, active vigorous: 51.1) and British women (inactive: 47.3, active vigorous: 50.4). In mental health functioning, the differences were generally smaller and not that clearly related to the intensity of physical activity. Emerging differences in health functioning were relatively small.

**Conclusions:**

Vigorous physical activity was associated with better subsequent physical health functioning in all three cohorts with varying magnitude. For mental health functioning, the intensity of physical activity was less important. Promoting leisure time physical activity may prove useful for the maintenance of health functioning among midlife employees.

**Strengths and limitations of this study**

- We used data from three occupational cohorts from Finland, Britain and Japan including women and men.
- A strength was also similarly measured, namely validated outcome of physical and mental health functioning.
- In addition, we used follow-up data considering key covariates in the analyses.
- Limitations include self-reported measures and differences in the measurement of leisure time physical activity.

## Introduction

Physical inactivity is a major public health problem worldwide.^1^ Physical inactivity is associated with poor physical as well as mental health functioning; however, the evidence comes mainly from cross-sectional studies.^2^ ^3^ Previous prospective studies, including the Nurses’ Health Study among US women,^4^ the Australian Longitudinal Study on Women's Health,^5^ the British Whitehall II (WHII) study^6^ and the Finnish Helsinki Health Study (HHS),^7^ have also shown that physical activity contributes to subsequent physical functioning already in midlife. There is evidence from the HHS that higher intensity physical activity is associated with better physical health functioning.^7^ Physical activity also contributes to mental health functioning; however, the association is less clear as for physical health functioning.^4^ ^5^ In addition, a previous HHS study suggests that, for mental health measured by psychotropic medication, the intensity of physical activity is not that important but increasing volume shows further benefits.^8^ A review concluded that avoiding inactivity is important for mental health.^9^

Finland, Britain and Japan are high-income countries that apart from similarities also share dissimilarities.^10^ From a public health perspective, these affluent societies differ.^11^ Leisure time physical activity patterns^12–14^ vary between these countries. In Scandinavia and in particular in Finland, participation in leisure time physical activity is relatively high, and in Britain somewhat lower.^12^ A recent study among 27 European countries^13^ also showed that the prevalence of physical inactivity during leisure time was the lowest in Finland (20%) and fairly high in the UK (40%). Also in Japan, leisure time physical activity is estimated to be low; only about one-fifth of midlife adults engage in 30 min or more of exercise two or more times per week.^14^ However, in Japan, the prevalence of overweight is very low^15^ compared with Britain and Finland where overweight among adults is relatively common.^16^ Furthermore, health behaviours such as smoking and alcohol use as well as socioeconomic patterns in health behaviours are different between these countries.^17^

It may well be that the association between physical activity and health vary between countries and cultures with different health behaviour and physical activity patterns. However, cross-cultural studies examining the association between physical activity and health in different countries are rare. The aim of this study was to examine whether leisure time physical activity contributes to subsequent physical and mental health functioning among midlife employees considering key covariates. The associations were tested in three occupational cohorts from Finland, Britain and Japan to show common and unique patterns.

## Methods

Prospective employee cohorts from the Finnish HHS (2000–2002 and 2007), British WHII I study (1997–1999 and 2003–2004) and Japanese Civil Servants Study (JACS) (1998–1999 and 2003) were used.

The baseline questionnaire surveys of the HHS were conducted in 2000, 2001 and 2002 among 40-year to 60-year-old employees of the City of Helsinki (n=8960, response rate 67%) (http://www.hjelt.helsinki.fi/hhs).^18^ The follow-up survey was conducted in 2007 among all the respondents of the baseline surveys (n=7332, response rate 83%). We included only white-collar employees excluding manual workers from the analyses to improve comparability to the WHII cohort. The analyses included 4986 women and 972 men from the HHS.

The WHII is a cohort of white-collar civil servants from 20 London-based civil service departments. At baseline (1985–1988), all participants were aged 35–55 years (n=10 308, response rate 73%) (http://www.ucl.ac.uk/whitehallII).^19^ The follow-up surveys have been conducted at 2.5-year intervals. We used phase 5 of data collection (1997–1999, response rate 76%) as the baseline and phase 7 (2003–2004, response rate 68%) as the follow-up. We included the participants who were in employment at wave 5 (65%) to improve the comparability with the other two cohorts, in which all were employed at the baseline. The analyses included 1064 women and 3078 men from the WHII study.

The baseline survey of the JACS was conducted in 1998–1999 among 20-year to 60-year-old local government employees working in a west coast province of Japan (n=4933, response rate 81%) (http://www.med.u-toyama.ac.jp/healpro).^20^ The follow-up survey (2003) included all those working at the time of the survey, whether or not they had participated in the baseline survey (n=4272, response rate 79%). We included only white-collar employees excluding manual workers in the analyses who were aged 35 years or over at baseline and had responded to both surveys. The analyses included 514 women and 1254 men from the JACS.

### Leisure time physical activity

In the HHS, the respondents were asked about their average weekly hours of physical activity or exercise during leisure time (commuting included) within the previous 12 months in four grades of intensity: walking, brisk walking, jogging and running, or their equivalent activities.^7^ In the WHII, a detailed questionnaire on leisure time different intensity physical activity/exercise was used to describe typical weekly physical activity.^21^ In the JACS, information on weekly hours of leisure time physical activity/exercise was asked in three intensity grades: light, moderate and vigorous. MET-hours per week were calculated for the respondents in all three cohorts using standardised MET values.^22^

Respondents in each cohort were classified into three ordinal groups taking into account the volume and intensity of physical activity: (1) inactive, (2) active moderate (meeting the moderate-intensity recommendation, eg, 2.5 h of brisk walking or equivalent activity per week) and (3) active vigorous (meeting the recommendation including vigorous physical activity, eg, jogging or equivalent activity for 45 min per week and brisk walking or equivalent activity for 1.5 h per week).^8^

### Physical and mental health functioning

Physical (PCS) and mental (MCS) component summary scores of the SF-36 health questionnaire were used as measures of physical and mental health functioning in this study. PCS and MCS are generic measures of physical and mental health functioning. The eight subscales of the SF-36 include physical functioning, role limitations due to physical problems, bodily pain, general health perceptions, mental health, role limitations due to emotional problems, social functioning and vitality. Each of these subscales positively or negatively contributes to both PCS and MCS. The PCS and MCS scores are continuous variables ranging from zero to 100 with a mean of 50 (SD=10) observed in the general US population. Low scores imply poor health functioning, while high scores imply good health functioning. The SF-36 has good construct validity and high test-retest reliability and internal consistency.^23^ ^24^

### Confounders

Confounders included age at the time of the baseline survey. Socioeconomic position (SEP) was measured by three occupational social classes for HHS and WHII: managers, professionals and clerical. For JACS, managers and professionals were combined.^17^ ^25^ Body mass index (BMI) was calculated by dividing the weight (kg) by the height (m) squared. Heavy drinking was dichotomised into heavy drinkers and those drinking less or abstainers. The cut-off point for heavy drinking was over 16 and over 24 units of alcohol per week among men and women, respectively.^26^ Smoking was dichotomised into non-smokers (including ex-smokers) and smokers following previous procedures.^10^ In addition, employment status at the follow-up was dichotomised into those working and those outside working life (eg, retirees and unemployed).

### Missing values

Those with missing values in the outcome, that is, PCS and MCS at the follow-up surveys or baseline leisure time physical activity, were excluded from the data. We used multiple imputation analyses for replacement of missing covariate values. In the HHS, the proportion of missing covariate values was low, with about 3% (n=200) of the participants having some missing values, whereas in the WHII the proportion of participants with some missing values was about 25% (n=996) and in the JACS it was even higher at 30% (n=525).

### Statistical analyses

The imputation analysis was conducted using the SPSS V.20. Ten imputed data sets were created and assumption of data missing at random was confirmed by the multiple imputation procedure. The estimates are obtained by averaging across the results from each of these 10 imputed data sets. We used a general linear model to calculate adjusted means and 95% CIs for PCS and MCS at follow-up by baseline physical activity groups. Statistically significant (p<0.05) differences between physical activity groups were indicated using the inactive as the reference group. All analyses were carried out separately for women and men in each cohort. In model 1, age was adjusted for. In model 2, age and SEP, BMI, heavy drinking, smoking and employment status at follow-up were adjusted for. In model 3, covariates in model 2 and baseline health functioning scores (PCS/MCS) were adjusted for after which the differences in mean scores reflect the emerging differences over the follow-up periods.

## Results

Description of baseline study variables are presented in table 1. Among the *Finnish* employees, one-fourth of women and men were considered inactive during their leisure time (table 1). Men were vigorously active more often than women. At baseline, the inactive had a physical health functioning mean score that was about four points lower than that of the vigorously active. The inactive also had lower scores of mental health functioning. Among the *British* employees, two-thirds of women and half of the men were considered inactive. Also among the British, men were vigorously active more often than women. The inactive women had an over three points lower physical health functioning score than did the vigorously active, whereas among the men the differences were smaller. In mental health functioning, the mean score differences were about two points, similar to the Finnish employees. Among the *Japanese* employees, a third of women and nearly half of the men were considered inactive during leisure time. Also among the Japanese, men were vigorously active more often than women. In physical health functioning, there were two point differences between physical activity groups among men and no differences among women. In mental health functioning, there were nearly three point differences between physical activity groups among women and men.

**Table 1:** Description of baseline study variables by physical activity groups

	Women			Men		
HHS	Inactive	Active moderate	Active vigorous	Inactive	Active moderate	Active vigorous
n (%)	1212 (24)	2258 (45)	1516 (30)	243 (25)	267 (27)	462 (48)
Age, M (SD)	50.0 (6.7)	49.7 (6.5)	47.7 (6.3)	51.5 (6.5)	52.4 (6.3)	49.6 (6.7)
SEP (%)						
Managers	32.2	27.3	36.1	54.7	57.7	65.2
Professional	18.9	21.4	25.1	29.6	25.5	24.2
Clerical	48.9	51.3	38.9	15.6	16.9	10.6
Alcohol (%)*	3.0	3.1	2.2	7.8	9.0	3.9
Smokers (%)	23.6	19.7	16.2	26.7	29.6	17.1
BMI, M (SD)	26.6 (5.0)	25.4 (4.2)	23.7 (3.4)	27.6 (4.9)	26.3 (3.6)	25.2 (3.0)
PCS, M (SD)	47.3 (9.1)	48.6 (8.5)	51.5 (7.1)	48.9 (7.8)	51.0 (6.9)	52.7 (5.8)
MCS, M (SD)	50.4 (10.8)	51.8 (9.7)	52.1 (8.9)	49.8 (11.4)	52.5 (8.8)	51.8 (9.5)
Working (%)†	64.6	67.5	79.6	63.4	57.7	72.7
**WHII**	**Women**			**Men**		
n (%)	748 (70)	134 (13)	182 (17)	1524 (50)	456 (15)	1098 (36)
Age, M (SD)	53.3 (4.9)	52.7 (4.9)	53.2 (4.8)	53.0 (4.7)	53.3 (4.9)	53.5 (5.2)
SEP						
Managers	25.0	29.1	28.0	36.0	36.9	37.4
Professional	42.0	50.0	54.9	58.5	60.0	59.3
Clerical	33.0	20.9	17.0	5.5	3.1	1.3
Alcohol (%)*	11.0	15.7	17.0	20.1	25.0	24.3
Smokers (%)	13.6	9.7	7.7	9.6	8.6	6.3
BMI, M (SD)	26.4 (4.8)	25.9 (4.9)	25.9 (4.8)	26.5 (3.8)	25.9 (3.6)	25.8 (3.3)
PCS, M (SD)	49.7 (8.6)	50.9 (6.8)	52.9 (6.5)	52.0 (6.5)	52.7 (6.3)	53.3 (5.7)
MCS, M (SD)	48.3 (10.4)	50.2 (8.9)	50.5 (8.8)	49.9 (9.0)	50.8 (9.5)	51.9 (8.2)
Working (%)†	68.0	71.6	62.1	72.0	75.7	73.2
**JACS**	**Women**			**Men**		
n (%)	181 (35)	288 (56)	45 (9)	571 (46)	407 (32)	276 (22)
Age, M (SD)	43.4 (7.5)	42.5 (6.9)	43.6 (6.3)	43.1 (7.1)	44.6 (7.4)	41.9 (7.2)
SEP						
Mana. and Prof	30.1	30.7	28.4	43.3	36.2	33.0
Clerical	69.9	69.3	71.6	56.7	63.8	67.0
Alcohol (%)*	5.0	6.9	13.3	15.1	22.9	20.3
Smokers (%)	5.5	4.9	4.4	46.4	46.4	32.2
BMI, M (SD)	21.8 (2.8)	21.9 (3.0)	22.1 (3.2)	23.4 (2.5)	23.6 (2.7)	23.3 (2.7)
PCS, M (SD)	48.6 (7.5)	49.2 (6.7)	49.1 (5.9)	50.0 (6.2)	50.9 (5.8)	51.2 (6.2)
MCS, M (SD)	44.8 (8.7)	45.6 (9.2)	47.0 (8.5)	46.0 (9.3)	46.9 (9.7)	47.3 (8.8)
Working (%)†	100.0	100.0	100.0	100.0	100.0	100.0

*Over 16 drinks/week for women and over 24 drinks/week for men.

†Working at the follow-up, in JACS, only those continuously employed were contacted at follow-up.

BMI, body mass index; HHS, Helsinki Health Study; JACS, Japanese Civil Servants Study; MCS, mental component summary SF-36; PCS, physical component summary SF-36; SEP, socioeconomic position; WHII, Whitehall II study.

### Leisure time physical activity and subsequent physical health functioning

#### Women

Among *Finnish* women, the vigorously active (49.5 points) had significantly higher mean scores of physical health functioning several years later than the inactive (46.0 points), whereas the moderately active (47.0 points) had only a somewhat higher score than the inactive in the age-adjusted model (table 2). After adjusting for other confounders, differences between physical activity groups attenuated but remained for the vigorous group. After adjusting for baseline physical health functioning statistical significance was lost; however, the patterns between physical activity groups remained, suggesting that some differences emerged during the follow-up between the inactive and vigorously active. Among the *British* women, the age-adjusted mean scores showed clear differences similar to the Finnish women. The vigorously active had significantly higher scores (50.4 points) than the inactive (47.3 points), whereas the moderately active (48.9 points) were in between. Adjusting for confounders attenuated the associations found but not to the same extent as among the Finnish women. After adjusting for baseline physical health functioning, no significant differences remained; however, the emerging differences in mean scores remained larger for the British than for the Finnish women. Among the *Japanese* women, the association was different; the inactive (47.1 points) and moderately active (47.0 points) tended to have higher scores of physical health functioning than the vigorously active (46.6 points), although the differences were not significant. Further adjustments for confounders and baseline physical health functioning had no effects.

**Table 2:** Physical health functioning mean scores (95% CI) at follow-up by baseline leisure time physical activity among women and men

Physical activity	N	Model 1	Model 2	Model 3
HHS women	4986			
Inactive	1212	46.0 (45.5 to 46.6)	46.8 (46.3 to 47.3)	47.4 (46.9 to 47.8)
Moderately active	2258	47.0 (46.6 to 47.3)*	47.2 (46.8 to 47.5)	47.3 (47.0 to 47.6)
Vigorously active	1516	49.5 (49.1 to 50.0)*	48.6 (48.2 to 49.1)*	47.9 (47.5 to 48.3)
WHII women	1064			
Inactive	748	47.3 (46.6 to 47.9)	47.4 (46.8 to 48.1)	47.7 (47.2 to 48.3)
Moderately active	134	48.9 (47.3 to 50.5)	48.6 (47.0 to 50.1)	48.5 (47.2 to 49.9)
Vigorously active	182	50.4 (49.0 to 51.7)*	50.0 (48.7 to 51.4)*	48.8 (47.6 to 50.0)
JACS women	514			
Inactive	181	47.1 (45.9 to 48.2)	47.0 (45.9 to 48.2)	47.2 (46.1 to 48.2)
Moderately active	288	47.0 (46.1 to 48.0)	47.0 (46.1 to 48.0)	47.0 (46.1 to 47.8)
Vigorously active	45	46.6 (44.2 to 49.0)	46.6 (44.3 to 49.0)	46.6 (44.4 to 48.8)
HHS men	972			
Inactive	243	47.8 (46.8 to 48.7)	48.6 (47.7 to 49.6)	49.5 (48.7 to 50.4)
Moderately active	267	49.7 (48.7 to 50.6)*	50.0 (49.1 to 50.8)*	50.0 (49.1 to 50.7)
Vigorously active	462	51.1 (50.4 to 51.8)*	50.5 (49.8 to 51.2)*	50.1 (49.5 to 50.7)
WHII men	3078			
Inactive	1524	50.3 (49.9 to 50.7)	50.5 (50.2 to 50.9)	50.8 (50.5 to 51.1)
Moderately active	456	50.5 (49.8 to 51.2)	50.3 (49.6 to 51.0)	50.3 (49.7 to 50.9)
Vigorously active	1098	51.2 (50.7 to 51.6)*	50.9 (50.5 to 51.4)	50.6 (50.2 to 51.0)
JACS men	1254			
Inactive	571	49.4 (48.8 to 49.9)	49.3 (48.8 to 49.9)	49.6 (49.1 to 50.1)
Moderately active	407	49.5 (48.9 to 50.2)	49.6 (48.9 to 50.2)	49.4 (48.7 to 50.0)
Vigorously active	276	50.8 (50.0 to 51.6)*	50.7 (49.9 to 51.5)*	50.5 (49.7 to 51.2)

Model 1: adjusted for age.

Model 2: adjusted for age, socioeconomic position, body mass index, smoking and alcohol use and employment status at follow-up.

Model 3: adjusted for age, socioeconomic position, body mass index, smoking and alcohol use, employment status at follow-up and baseline physical health functioning.

*Significantly (p<0.05) different from the inactive.

HHS, Helsinki Health Study; JACS, Japanese Civil Servants Study; WHII, Whitehall II study.

#### Men

Among the *Finnish* men, the vigorously active (51.1 points) and the moderately active (49.7 points) had significantly higher mean scores than the inactive (47.8 points) in the age-adjusted model. After adjusting for confounders, differences between physical activity groups attenuated but remained significant. Adjusting for baseline physical health functioning further attenuated the differences and no significant differences emerged; however, the patterns remained. Among the *British* men, the age-adjusted mean scores showed relatively small differences compared with the Finnish men, although the vigorously active (51.2 points) had significantly higher mean scores than the inactive (50.3 points). After adjusting for confounders, no significant differences remained and adjusting for baseline physical health functioning further attenuated the differences and no clear patterns remained. Among the *Japanese* men, the vigorously active (50.8 points) had significantly higher mean scores than the inactive (49.3 points). Adjusting for confounders slightly attenuated the associations, but significant differences remained. After adjusting for baseline physical health functioning, significant differences were lost; however, the emerging differences remained clearer than for the Finnish and British men.

### Leisure time physical activity and subsequent mental health functioning

#### Women

In mental health functioning, among the *Finnish* women*,* the moderately (52.3 points) and vigorously active (52.4 points) had significantly higher mean scores than the inactive (51.0 points) in the age-adjusted model (table 3). Adjusting for confounders had no effects. Adjusting for baseline mental health functioning attenuated the associations found; however, the patterns between physical activity groups and some significant differences remained, suggesting that differences in mental health functioning emerged during the follow-up. Among the *British* women, the associations were clearer than among the Finnish women in the age-adjusted model, although only the vigorously active (52.6 points) had significantly higher mean scores than the inactive (50.4 points). Adjusting for confounders had no effect on the associations found. Adjusting for baseline mental health functioning attenuated the associations further, however, the emerging differences were larger than for the Finnish women. Among the *Japanese* women, the age-adjusted mean scores were notably lower than for the Finnish and British women. The vigorously active women (46.7 points) had higher scores than the moderately active (44.5 points) and inactive (43.5 points), although they were statistically non-significant. Adjusting for confounders had no effect on the differences. Adjusting for baseline mental health functioning attenuated the associations further; however, the emerging differences were larger than for the Finnish and British women.

**Table 3:** Mental health functioning mean scores (95% CI) at follow-up by baseline leisure time physical activity among women and men

Physical activity	N	Model 1	Model 2	Model 3
HHS women	4986			
Inactive	1212	51.0 (50.4 to 51.6)	51.0 (50.5 to 51.6)	51.6 (51.1 to 52.1)
Moderately active	2258	52.3 (51.8 to 52.7)*	52.3 (52.0 to 52.5)*	52.2 (51.8 to 52.6)*
Vigorously active	1516	52.4 (51.9 to 52.9)*	52.3 (51.8 to 52.9)*	52.1 (51.6 to 52.5)
WHII women	1064			
Inactive	748	50.4 (49.7 to 51.0)	50.4 (49.7 to 51.0)	50.6 (50.0 to 51.2)
Moderately active	134	52.0 (50.4 to 53.6)	52.0 (50.4 to 53.6)	51.5 (50.0 to 53.0)
Vigorously active	182	52.6 (51.2 to 54.0)*	52.6 (51.2 to 53.9)*	52.0 (50.7 to 53.2)
JACS women	514			
Inactive	181	43.5 (42.0 to 45.0)	43.5 (42.0 to 45.0)	43.8 (42.3 to 45.2)
Moderately active	288	44.5 (43.3 to 45.7)	44.5 (43.3 to 45.7)	44.4 (43.3 to 45.6)
Vigorously active	45	46.7 (43.6 to 49.7)	46.7 (43.6 to 49.8)	46.2 (43.3 to 49.1)
HHS men	972			
Inactive	243	52.1 (50.9 to 53.3)	52.1 (50.9 to 53.3)	53.0 (51.9 to 54.1)
Moderately active	267	51.9 (50.7 to 53.1)	52.2 (51.5 to 52.8)	51.8 (50.7 to 52.8)
Vigorously active	462	52.5 (51.6 to 53.3)	52.3 (51.4 to 53.2)	52.1 (51.3 to 52.9)
WHII men	3078			
Inactive	1524	51.5 (51.1 to 51.9)	51.5 (51.1 to 51.9)	51.9 (51.5 to 52.2)
Moderately active	456	52.0 (51.2 to 52.8)	52.0 (51.2 to 52.8)	52.0 (51.3 to 52.7)
Vigorously active	1098	52.9 (52.4 to 53.4)*	52.9 (52.4 to 53.4)*	52.4 (52.0 to 52.8)
JACS men	1254			
Inactive	571	46.6 (45.8 to 47.4)	46.6 (45.8 to 47.4)	46.9 (46.1 to 47.6)
Moderately active	407	47.6 (46.7 to 48.6)	47.6 (46.7 to 48.5)	47.6 (46.7 to 48.4)
Vigorously active	276	47.8 (46.7 to 48.9)	47.7 (46.6 to 48.9)	47.4 (46.3 to 48.4)

Model 1: adjusted for age.

Model 2: adjusted for age, socioeconomic position, body mass index, smoking and alcohol use, and employment status at follow-up.

Model 3: adjusted for age, socioeconomic position, body mass index, smoking and alcohol use, employment status at follow-up and baseline mental health functioning.

*Significantly (p<0.05) different from the inactive.

HHS, Helsinki Health Study; JACS, Japanese Civil Servants Study; WHII, Whitehall II study.

#### Men

Among the *Finnish* men, there were no significant differences in mental health functioning between physical activity groups (table 3), whereas, among the *British* men, age-adjusted mean scores showed significant differences between the inactive (51.5 points) and the vigorously active (52.9 points). Adjusting for confounders had no effect on the associations found. Adjusting for baseline mental health functioning attenuated the associations and no significant differences emerged, although the patterns remained. Also among the *Japanese* men, the age-adjusted mean scores of mental health functioning were clearly lower than for the Finnish and British men. The vigorously active (47.8 points) and moderately active (47.6 points) had higher mean scores than the inactive (46.6 points), although they were statistically non-significant. Adjusting for confounders had no effect on the differences. Adjusting for baseline mental health functioning further attenuated the associations.

## Discussion

Leisure time physical activity was associated with better subsequent physical health functioning in all three examined cohorts; however, the magnitude of the associations varied and some gender differences were found. The clearest differences were found in subsequent physical health functioning among the Finnish women and men and British women, suggesting that the vigorously active have better physical health functioning. In mental health functioning, the differences by physical activity were generally smaller than for physical health functioning. The emerging differences in physical and mental health functioning during the follow-up were of similar magnitude and relatively small.

The benefits of vigorous activity on physical health functioning were evident in all three cohorts, although clinically meaningful differences (>3 points)^27^ were observed only among the Finnish women and men as well as the British women. However, for the general population, there is no consensus on the magnitude of a clinically meaningful difference.^3^ A previous study, with the Finnish data, suggested that those vigorously active have better physical health functioning than those moderately active with the same total amount of leisure time physical activity.^7^ The benefits of vigorous activity on physical health functioning are also supported by another earlier prospective study that followed a cohort of Finnish industrial employees for 28 years.^28^ Some studies have shown the importance of physical fitness on functioning.^29^ Our findings relating to the benefits of vigorous activity compared to moderate activity may also reflect increased fitness. Muscle strength training, especially, is considered a particularly important part of exercise in intervention studies.^30^ However, in these data, it is not possible to examine muscle strengthening exercises separately as the questionnaires do not allow it.

Previous prospective studies have suggested weaker associations between physical activity and mental health functioning than for physical aspects of health functioning,^5^ ^6^ which is partly in line with our study. Those vigorously active had better mental health functioning, although the differences were not as clear as for physical health functioning and, in addition, moderate-intensity physical activity also had beneficial effects on mental health functioning. The results of our study are in accordance with previous studies, suggesting that the intensity of physical activity is not that important for mental health.^8^ ^9^ An intervention study from Australia showed that a combined aerobic and weight-training exercise improves the mental health functioning of employees.^31^

Good physical and mental health functioning is vital for work ability and participation in everyday life. Poor physical as well as mental health functioning (SF-36) has been shown to predict longer sickness absence in the Finnish cohort^32^ and even mortality.^33^ These self-reported general measures of physical and mental health functioning capture a wide range of health problems that affect health functioning in everyday life and are thus important markers of health. In midlife, limitations in functioning are usually related to chronic diseases and conditions^34^ such as musculoskeletal diseases and mental disorders. Physical activity is associated with reduced risk of various musculoskeletal diseases such as neck and low back disorders and arthrosis,^35^ which directly influence physical functioning. Regular physical activity is also associated with a reduced risk of mental disorders such as depression and anxiety.^36^

Although there were similarities across countries, one important contribution of this paper was to point out that the associations also differed somewhat between the three cohorts. One previous study suggests that physical activity has similar associations with mortality in the French GAZEL and British WHII cohorts.^37^ In our study, the associations were quite similar for the Finnish and British women, whereas among the Japanese women the associations were found only for mental health functioning. Among men, there were more notable differences between the cohorts as in contrast to the British and Japanese men, among the Finnish men no differences in mental health functioning were found between physical activity groups. In a previous study within the same Finnish cohort, higher physical activity was, however, associated with lower subsequent psychotropic medication purchases, suggesting an association with better mental health.^8^ In addition, it should be noted that the adjustment made for key covariates attenuated the found associations more clearly among the Finnish women and men than among the British and Japanese employees. Differences in the distribution of covariates between the cohorts might have contributed to this as, for example, BMI differences by physical activity groups tended to be clearer among the Finnish employees. Since there are clear differences in the prevalence of health behaviours and BMI between these countries as well as among women and men, it would be intriguing to examine, for example, whether there are differences in the joint associations of BMI and physical activity with health in these cohorts.

There were notable differences in physical activity patterns between countries in this study. About a quarter of the Finnish women and men were considered inactive, whereas among the British physical inactivity was clearly higher as half of the men and even more women were considered inactive. This largely follows the physical activity patterns reported in previous studies.^12^ ^13^ Among the Japanese, a third of the women and nearly half of the men were considered inactive. Vigorous activity was most common among the Finnish and least common among the Japanese. Some similarities were also observed in all three study cohorts as men were vigorously active more often than women, which is in accordance with previous studies.^38^

Adjusting for the baseline health functioning score in follow-up studies has been criticised as it may be problematic methodologically and lead to biased results if the baseline score is associated with the exposure.^39^ ^40^ In our study, physical activity was associated with physical and mental health functioning scores at the baseline measurement. We adjusted for PCS and MCS baseline scores in the last model in which the mean score differences between physical activity groups should be interpreted as emerging differences over the follow-up period. The baseline score adjusted differences between physical activity groups were relatively small; in other words, the differences between physical activity groups remained largely the same over the follow-up. However, some differences emerged over the follow-up, suggesting that physical activity has a positive effect on physical as well as mental health functioning.

### Strength and limitations

We examined three occupational cohorts from high-income countries: Finland, Britain and Japan. The data collecting procedure was harmonised to a large extent including similarly measured outcome and covariates. The SF-36 is a well-known health measure used and validated in different countries,^41^ ^42^ offering a self-reported general measure of physical and mental health that has also been used in previous studies.^24^ We were also able to take key confounders into account in all three cohorts. Absence of data on non-communicable conditions is a potential limitation and could lead to confounding in the associations. However, we considered baseline health status by adjusting for the baseline health functioning. Furthermore, according to our sensitivity analyses, adjusting for any limiting long-standing illness at baseline produced similar results (data not shown). There are also other limitations such as the use of somewhat different physical activity questionnaire batteries for measuring leisure time physical activity. Although no physical activity questionnaire has proven superior,^43^ the use of the same validated questionnaire in each cohort would have strengthened the comparability between cohorts. Slightly different MET values were applied in each cohort; however, it was considered in the classification, that is, in each cohort, MET-hours per week comparable to 2.5 h of brisk walking or equivalent moderate-intensity activity were used as the cut-point for being active. In general, these measures are to capture the same phenomena, that is, leisure time physical activity, but direct comparisons between levels of physical activity are not warranted. Since our focus was on the association between leisure time physical activity and subsequent health functioning, differences in the measurement of physical activity are less crucial. Nonetheless, caution should be applied when comparing the results. Further studies examining the association between physical activity and health in different cohorts should use objective physical activity measurements to improve comparability. When the outcome is also self-reported, same source bias may exist. However, physical activity has shown similar associations with register-based general health measures such as disability retirement^44^ and mortality^21^ ^45^ in these data. In addition, there was some missing information on study variables which might cause bias to the examined associations. Therefore, we used multiple imputation to correct for the possible bias due to missing values of the covariates. We also made complete case analyses (data not shown) and the associations were broadly similar, suggesting only a minor bias due to missing values. In addition, it should be noted that the data sets are not the most recent; however, using similar time frames from each study is important for the comparability between the cohorts. Furthermore, while, for example, working conditions have changed during the past decades,^46^ possibly contributing to this association, it is unlikely that the association between physical activity and health functioning has changed within the past decade.

## Conclusions

We examined whether leisure time physical activity contributes to subsequent physical and mental health functioning among midlife employees from Finland, Britain and Japan. Vigorous physical activity was associated with better physical health functioning several years later among the Finnish, British and Japanese employees, however, with varying magnitude and some gender differences. For mental health functioning, the intensity of physical activity appeared less important. Nonetheless, when motivating people to participate in regular physical activity, a focus on functioning is likely to prove useful as it relates to people's everyday lives and covers a large number of midlife people. Promoting leisure time physical activity among midlife employees may prove useful for maintaining health functioning and work ability.
